# Hypertrophic Pyloric Stenosis in a Four-Week-Old Infant: Radiological Diagnosis and Pitfalls

**DOI:** 10.7759/cureus.98606

**Published:** 2025-12-06

**Authors:** Biraj Pokhrel, Aaraju Bhatta, Anil Basnet, Niraaz Pokhrel, Dosti Regmi

**Affiliations:** 1 Department of General Medicine, Prithvi Chandra Hospital, Ramgram, NPL; 2 Department of Medicine, Tribhuvan University Institute of Medicine, Kathmandu, NPL; 3 Department of Internal Medicine, Patan Academy of Health Sciences, Lalitpur, NPL; 4 Department of Medicine, Shahid Gangalal National Heart Centre, Kathmandu, NPL; 5 Department of Radiology, Nepalese Army Institute of Health Sciences, Kathmandu, NPL; 6 Department of Pediatric Radiology, Le Bonheur Children's Hospital, Memphis, USA

**Keywords:** gastric outlet obstruction, hypertrophic pyloric stenosis, infant, pyloromyotomy, ultrasonography

## Abstract

Infantile hypertrophic pyloric stenosis (IHPS) is a common acquired condition of infancy, characterized by pyloric muscle hypertrophy leading to complete or near-complete gastric outlet obstruction. A typical presentation includes projectile, non-bilious vomiting. While the palpable "olive" and visible peristaltic waves are classic signs, ultrasonography is the diagnostic modality of choice due to its high accuracy. Diagnosis relies on established sonographic thresholds for pyloric muscle thickness and canal length. Differential diagnoses include transient pylorospasm, which resolves on its own, and prostaglandin-induced mucosal hypertrophy, where only the muscular wall should be measured. Herein, we present the case of a four-week-old male infant with projectile non-bilious vomiting and failure to thrive. Ultrasonography confirmed the diagnosis, demonstrating the classic radiological signs. The patient was successfully managed with fluid resuscitation and a Ramstedt pyloromyotomy. This case highlights the pivotal role of ultrasonography in diagnosing IHPS, discusses relevant differential diagnoses, and underscores the importance of recognizing potential imaging pitfalls, such as a posteriorly displaced pylorus due to an overdistended stomach.

## Introduction

Infantile hypertrophic pyloric stenosis (IHPS) is a condition affecting young infants, usually in the second to 12th weeks of life, characterized by abnormal thickening of the pyloric musculature in the stomach [1,2]. This hypertrophy leads to a progressive, near-complete gastric outlet obstruction. While the precise etiology remains elusive, IHPS is understood to have a multifactorial origin, incorporating genetic predispositions, hormonal influences, neurogenic factors, and environmental triggers such as maternal macrolide exposure (e.g., erythromycin), smoking, and bottle feeding [3-5]. This condition demonstrates a strong male predominance, with a reported male-to-female ratio of approximately 5:1 [2].

Timely diagnosis is paramount to facilitate prompt intervention and prevent complications such as dehydration and electrolyte imbalances. In this context, ultrasonography has emerged as the diagnostic modality of choice. The literature robustly supports the diagnostic accuracy of key sonographic measurements, including pyloric muscle thickness, channel length, and pyloric diameter, for confirming IHPS [6]. While an abdominal radiograph may occasionally reveal a "caterpillar sign," this finding is not consistently present, limiting its reliability [7].

Herein, we present a case of IHPS, with a primary focus on its definitive radiologic evaluation and an analysis of common interpretive challenges to aid in accurate diagnosis. This report is prepared in accordance with CARE-radiology criteria [8].

## Case presentation

**Figure 1:**
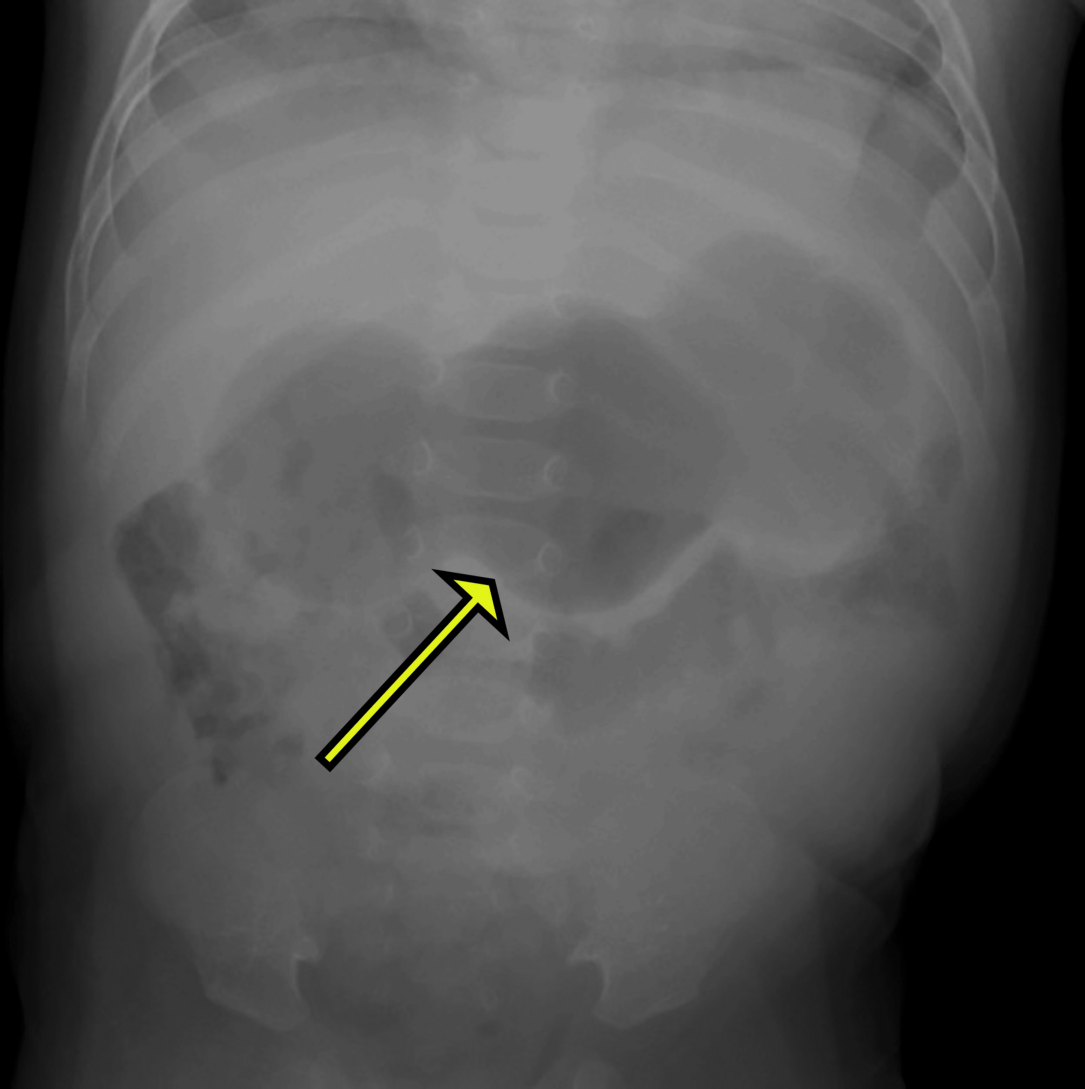
AP view radiograph of the abdomen shows a peristaltic dilated stomach giving the classic caterpillar sign (yellow arrow). AP, Anterior-Posterior view

A four-week-old full-term male infant presented to the emergency department with a history of projectile, non-bilious vomiting occurring after feeds. The parents also reported decreased weight gain. Anteroposterior abdominal radiograph showed a distended stomach with hyperperistalsis, creating the classic "caterpillar sign" (Figure 1).

**Figure 2:**
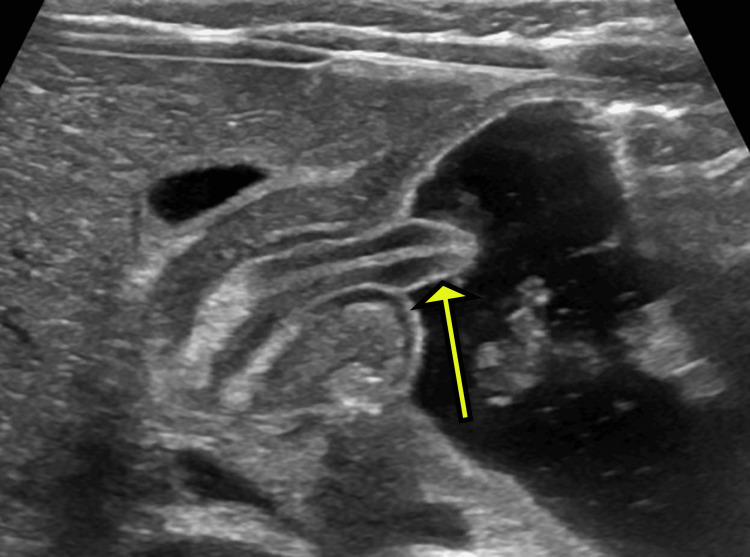
Longitudinal ultrasonography of the antropyloric region shows that the antrum is distended by fluid and the mucosa from the pylorus is squeezed out into the antrum, giving the "nipple" appearance (yellow arrow).

**Figure 3:**
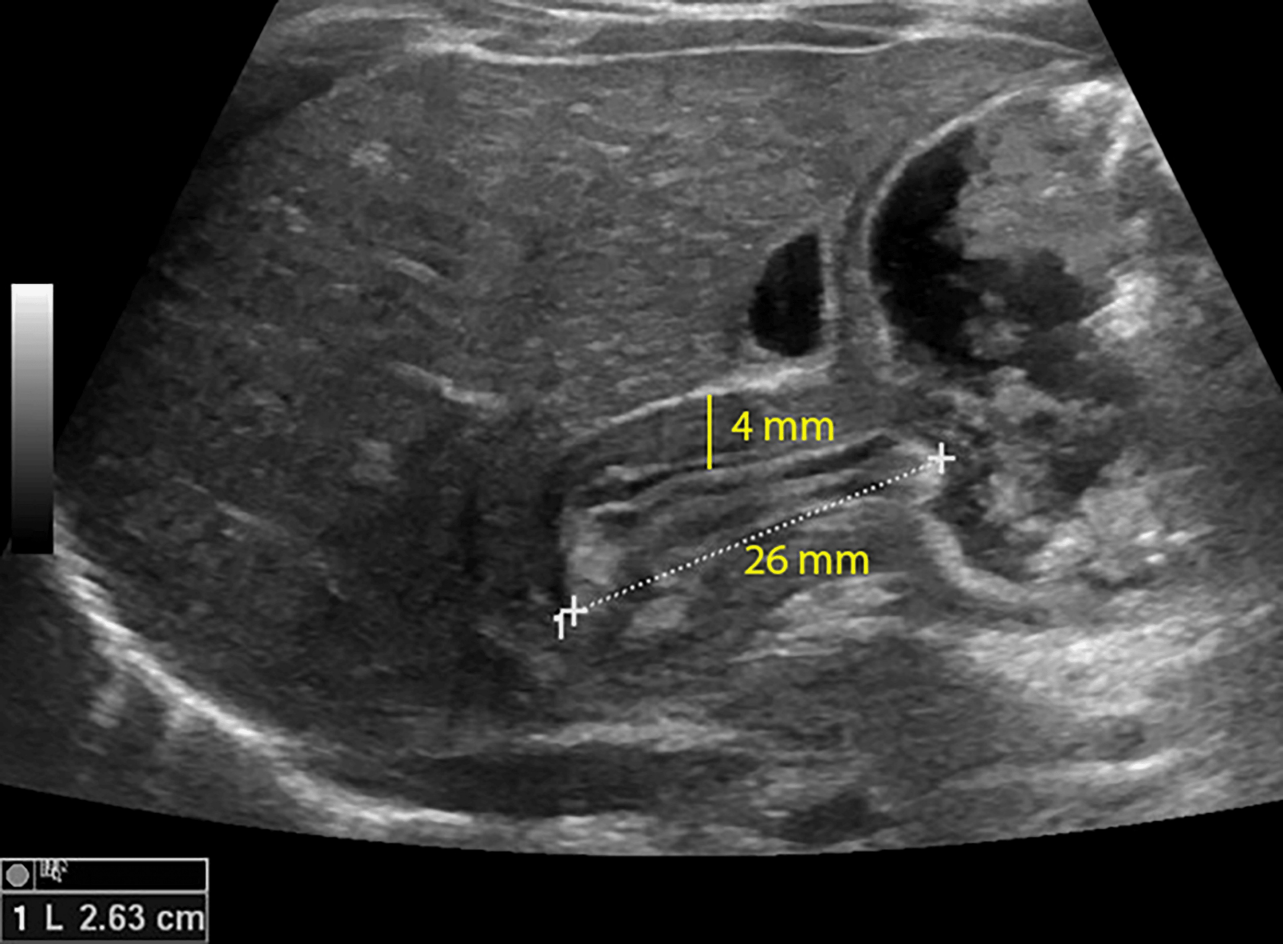
Longitudinal ultrasonography of the antropyloric region shows that an elongated and thickened pylorus spanning a length of 26 mm (white dotted line) and has a hypoechoic muscular wall measuring 4 mm in thickness (yellow line), which fulfills the criteria for the diagnosis of IHPS. IHPS, Infantile Hypertrophic Pyloric Stenosis

Longitudinal ultrasonography of the antropyloric region revealed a thickened hypoechoic pyloric muscle with a wall thickness of 4 mm and a canal length of 24 mm. The distended antrum and mucosal protrusion into the antrum gave rise to the "nipple sign" (Figures 2, 3).

**Figure 4:**
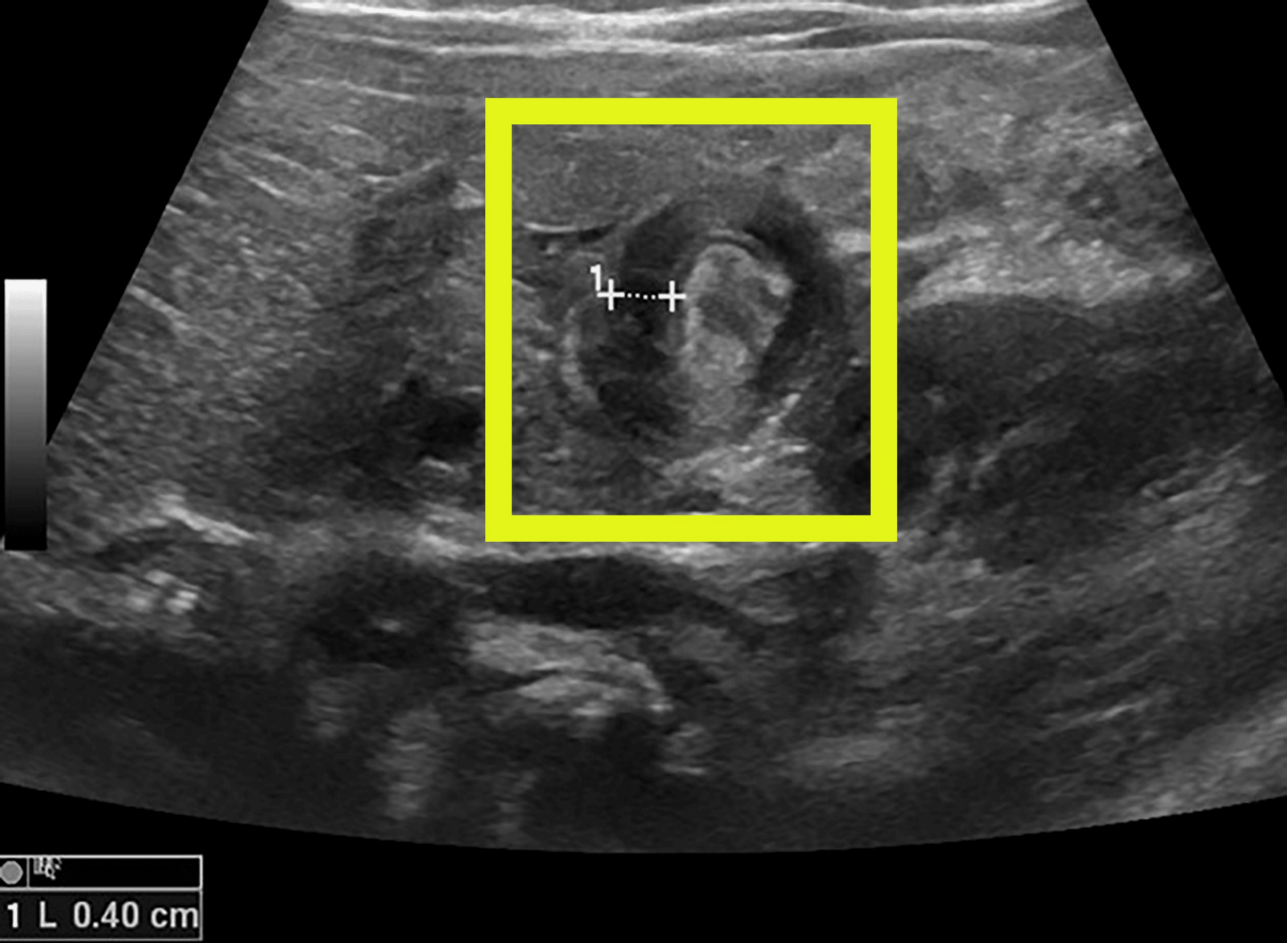
Transverse ultrasonography through the pylorus (yellow box) shows a thickened hypoechoic pyloric muscular wall and central echogenic mucosa, a typical “doughnut” appearance in IHPS. IHPS, Infantile Hypertrophic Pyloric Stenosis

No relaxation of the pylorus was noted during the scan, and the pylorus maintained a constant appearance. Transverse ultrasonography showed a concentric thickened hypoechoic muscular wall with central echogenic mucosa, producing the classic "doughnut sign" (Figure 4).

The findings met the diagnostic criteria for IHPS. The patient underwent fluid and electrolyte correction, followed by a successful Ramstedt pyloromyotomy. The postoperative period was uneventful. The patient's electrolyte imbalance was corrected, and he was discharged the following day.

## Discussion

The pylorus, functioning as the stomach's gatekeeper, is an anatomically complex sphincter. It is formed by two circular muscle loops connected by a longitudinal muscle fiber tract, which is innervated by sympathetic nerves. It prevents the premature entry of undigested food into the small intestine and also protects against the reflux of stomach contents into the small intestine when the small intestine contracts. In IHPS, there is progressive hypertrophy of the pylorus, which causes near-complete obstruction of the gastric outlet and lengthening of the pyloric canal. The pathophysiology is multifactorial and thought to involve a complex dysfunction involving the enteric nervous system, parietal cells, gastrointestinal hormones, and interstitial cells of Cajal. Key abnormalities include disrupted hormonal control, extracellular matrix defects, smooth muscle dysfunction, altered growth factors (such as increased insulin-like growth factor), and impaired nitric oxide signaling. This results in failure of muscle relaxation coupled with increased synthesis of growth factors, ultimately leading to muscle hyperplasia, hypertrophy, and obstruction [9,10].

IHPS is an acquired condition that manifests during the second to 12th weeks of infancy. Classically, they present with forceful, projectile, non-bilious vomiting. Hypertrophy of the pylorus can sometimes be palpated, especially when the stomach is emptied or decompressed by using a nasogastric tube, as a firm, non-tender, hard mass, which is described as the "olive sign." A peristaltic wave can be seen in thin patients, traveling from the left upper quadrant and across the epigastrium. If not treated, subsequently, dehydration (signs: depressed fontanelles, dry mucous membranes, decreased tearing, poor skin turgor, and lethargy) and metabolic disturbance follow. The electrolyte imbalance that is seen is hypochloremic, hypokalemic metabolic alkalosis. Hypernatremia or hyponatremia may succeed dehydration, leading to prerenal renal failure [6,11].

Early diagnosis is crucial, and imaging plays a vital role, as palpation has limited sensitivity. Abdominal radiograph, though non-specific, can sometimes catch the hyperperistalsis of the distended stomach with the typical caterpillar sign. Ultrasonography is a highly sensitive (97%), specific (100%), and accurate method for the diagnosis of IHPS [9]. The findings are best visualized with the stomach distended. Hence, oral electrolyte solution or milk can be given before the study. The hypertrophied muscle of the pylorus appears hypoechoic in contrast to the liver and is usually seen adjacent to the gall bladder. The measurement of the canal length and muscular wall is obtained.

Hypertrophic pylorus shows the following features [12]: (a) canal length greater than 16 mm; (b) diameter usually greater than 11 mm; (c) circular muscle usually more than 2.5 mm thick; (d) shouldering of the ends of the pyloric canal; (e) canal does not open; (f) little passage of gastric contents; and (g) increased gastric peristalsis.

It is important to note that these thresholds may be adjusted for premature or younger neonates, where smaller measurements can still be diagnostic [13]. The literature also reports supporting findings, such as the antral nipple sign and the double-track sign, observed on ultrasonography; however, current evidence is insufficient to evaluate their usefulness and diagnostic accuracy [14,15].

According to American College of Radiology (ACR) guidelines, contrast upper gastrointestinal (fluoroscopic UGI) contrast studies can also be used to evaluate obstructive causes of vomiting. In IHPS, this study may show a "string sign," where only a thin thread of contrast passes through the narrowed, elongated pyloric channel. However, fluoroscopy is less favored due to its time-consuming nature, inherent radiation exposure, and user-dependent sensitivity [16].

Imaging differential diagnosis

Pylorospasm

The transient spasm of the pylorus can mimic an IHPS. The transient spasm resolves and shows peristalsis, but the IHPS never changes its appearance, and peristalsis is never seen. A follow-up scan is suggested if suspicion arises.

Prostaglandin-Induced Mucosal Foveolar Hypertrophy

Prostaglandin therapy given to children with cardiac conditions can cause mucosal hypertrophy and decreased contraction in the GI tract. The non-contracted pylorus with a lumen filled with hypertrophied mucosa can cause increased pyloric diameter and length. Hence, the hypoechoic muscular wall alone should be measured. Other causes of mucosal hypertrophy include mild allergy, granulomatous disease, or eosinophilic gastroenteritis [17].

Pitfall 1

When the stomach is overtly distended, the hypertrophied pylorus may also be displaced posterior to the antrum, leading to false-negative results. Turning the child to the right lateral decubitus position displaces the fluid to the fundic side, and the pylorus moves anteriorly, allowing it to be well visualized [10].

Pitfall 2

Occasionally, the cephalic angulation of the probe can show the gastroesophageal junction, and the reflux from the distended fundus of the stomach to the esophagus could be mistaken for a normally peristaltic pylorus.

Although the literature mentions the non-surgical treatment with atropine sulfate, the mainstay of treatment is surgical, with preoperative resuscitation. After correcting electrolyte imbalances and dehydration, a Ramstedt pyloromyotomy is performed. Prognosis is excellent with timely intervention [18,19].

## Conclusions

IHPS remains a crucial differential diagnosis in infants with projectile vomiting. Radiological findings, especially on ultrasonography, are diagnostic and should be promptly pursued. Awareness of diagnostic pitfalls and differential conditions is essential for accurate assessment. Early diagnosis and surgical intervention result in excellent outcomes.
